# Animal Models for Hepatitis E Virus

**DOI:** 10.3390/v11060564

**Published:** 2019-06-18

**Authors:** Laura Corneillie, Dominic H. Banda, Philip Meuleman

**Affiliations:** Laboratory of Liver Infectious Diseases, Department of Diagnostic Sciences, Faculty of Medicine and Health Sciences, Ghent University, 9000 Ghent, Belgium; laura.corneillie@ugent.be (L.C.); dominic.banda@ugent.be (D.H.B.)

**Keywords:** Hepatitis E virus, animal model, zoonosis, cross-species infection, pathogenicity, vaccine

## Abstract

Hepatitis E virus (HEV) is an underdiagnosed pathogen with approximately 20 million infections each year and currently the most common cause of acute viral hepatitis. HEV was long considered to be confined to developing countries but there is increasing evidence that it is also a medical problem in the Western world. HEV that infects humans belongs to the *Orthohepevirus A* species of the *Hepeviridae* family. Novel HEV-like viruses have been observed in a variety of animals and some have been shown to be able to cross the species barrier, causing infection in humans. Several cell culture models for HEV have been established in the past years, but their efficiency is usually relatively low. With the circulation of this virus and related viruses in a variety of species, several different animal models have been developed. In this review, we give an overview of these animal models, indicate their main characteristics, and highlight how they may contribute to our understanding of the basic aspects of the viral life cycle and cross-species infection, the study of pathogenesis, and the evaluation of novel preventative and therapeutic strategies.

## 1. Introduction

Hepatitis E virus (HEV) was discovered in the early 1980s as a causative agent of non-A, non-B hepatitis (NANB), and is currently one of the major causes of acute viral hepatitis worldwide [1]. It is a quasi-enveloped, single-stranded RNA virus with a positive-sense genome that is approximately 7.2 kb in size [2]. The viral genome comprises three open reading frames (ORF 1–3), which are flanked by 5′ and 3′ untranslated regions (UTR) [2]. At the end of the 5′ UTR a 7-methylguanylate (m^7^G) cap is present, which drives cap-dependent genome translation. The 3′ UTR, which is about 65 nucleotide long, possesses a poly-adenylated tail [2]. ORF1 encodes a 1693-amino-acid-long nonstructural polyprotein, which is comprised of functional domains that are essential for viral genome replication [3,4]. There is still controversy and uncertainty regarding whether this polyprotein is proteolytically processed following translation, as is the case for other positive-strand RNA viruses [5]. ORF2 and ORF3, which are overlapping, are encoded from a bicistronic subgenomic RNA [6]. ORF2 encodes the viral capsid protein, which encapsulates the viral genome. The capsid protein possesses three domains: the S (shell), M (middle), and P (protruding) domains. The M and P domains are believed to play vital roles during virus entry [7]. More recently, it was shown that ORF2 also encodes a secreted form (ORF2^S^), for which the biological role needs to be further elucidated. In cell culture, ORF2^S^ had the ability to reduce antibody-mediated translation [8,9]. ORF3 encodes a phosphoprotein that plays an important role during viral particle morphogenesis [10].

HEV is classified into the family of the *Hepeviridae*. There are two recognized genera under this family, namely the *Orthohepevirus*, which comprises all mammalian and avian HEV isolates, and the *Piscihepevirus*, which comprises the closely related cutthroat trout virus [11,12]. The genus *Orthohepevirus* encompasses four species, *Orthohepeviruses A* to *D.* Classification into species is based on host range and sequence identity [6].

*Orthohepevirus A* consists of 8 major genotypes. Genotypes 1 and 2 can solely infect humans. Isolates from these genotypes are primarily transmitted via fecal-oral route, thereby causing waterborne hepatitis outbreaks in regions of poor sanitation in Asia, Africa, and Central America, with an estimated amount of 20 million infected people each year [13]. Infections mostly result in a self-limiting acute icteric hepatitis. However, in certain patient groups, it is associated with progressive liver disease, leading to fulminant hepatitis and death. Mortality rates are generally low but increase in infants under 2 years and reach 25% in pregnant women [14,15].

Genotype 3 and 4 HEV are responsible for autochthonous infections in developed countries. Genotype 3 has been isolated from pigs, wild boar, rabbits, deer, mongooses, and rats. However, rats are not the main hosts for genotype 3 HEV, but rather for specific rat HEV belonging to the *Orthohepevirus B* species, as stated below. Genotype 3 HEV has been isolated from rats in the United States, but could not be retrieved from Eastern European rats, so further confirmation is needed [16,17]. Genotype 4 HEV has been reported to be predominantly found in domestic and wild swine, as well as in sheep [5]. Infections with these genotypes are mainly zoonotic, via consumption of undercooked contaminated animal products [6]. However, other transmission routes have also been described. HEV RNA was previously detected in leafy green vegetables and on-field grown strawberries, which probably originated from use of contaminated irrigation or surface water [18,19]. Coastal waters can also become contaminated with HEV via polluted rivers or sewage water, leading to the presence of the virus in shellfish [20]. Shellfish consumption is a risk factor for acquiring HEV, as these are mostly eaten raw or only slightly cooked. Shellfish have been implicated as a viral source in several European case reports, as well as for an HEV outbreak on a cruise ship [21,22,23,24]. Moreover, studies in the United Kingdom have shown a coastal clustering of HEV, as an increase in patients is observed within two kilometers of the sea [22,25]. The exact reason remains to be clarified but could be related to increased consumption of shellfish and environmental factors, such as flooding in the area leading to increased exposure of waterborne pollutants [25]. 

Direct contact with infected animals may also lead to HEV, as higher seroprevalence rates occur in swine workers, veterinarians, and pork butchers. Additionally, blood-borne transmission has been described [26]. Infections with genotype 3 and 4 mostly resolve spontaneously, but chronic HEV may occur in immunocompromised persons, such as organ transplant recipients, persons with hematological malignancies, HIV-infected individuals, and rheumatological patients [27,28]. 

HEV of Genotype 5 and 6 have only been described in wild boar, whereas isolates from camels are classified into genotype 7 and 8 [11]. Interestingly, camel HEV (genotype 7) was isolated from a patient in the Middle East who had regularly been consuming camel meat and milk, and may therefore also pose a risk for zoonotic infection [4]. 

Beside the abovementioned animals, anti-HEV antibodies have also been detected in dogs, cats, cattle, goats, and horses, without knowing the source of seropositivity [29]. For horses in particular, independent studies in China, Egypt, and Spain demonstrated a seroprevalence ranging from 0.8% to 16.6% [30,31,32]. Phylogenetic analyses showed a close relationship with previously described human and swine HEV. Further research is needed to verify if horses are true reservoirs or rather spillover hosts [30].

Next to HEV, isolates belonging to the *Orthohepevirus A* species, a variety of HEV-like viruses, have been identified in other animal species. Isolates from chickens belong to *Orthohepevirus B*, whereas isolates from rats, greater bandicoots, Asian musk shrews, ferrets, and mink belong to *Orthohepevirus C*. Isolates from bats belong to *Orthohepevirus D* [33]. Recently, a 56-year-old Chinese liver transplant patient was reported to have been infected by rat HEV and the infection manifested as a persistent hepatitis, highlighting the potential of rat HEV to cause zoonosis [34].

Although self-limiting in immunocompetent individuals, HEV still poses a global health burden with a potential threat to food safety, especially given the ever expanding range of HEV and HEV-like agents. Efforts must, thus, be undertaken to better understand the nature of this pathogen [35].

Just like other hepatitis viruses, HEV is tremendously difficult to propagate in cell culture. However, recent breakthroughs in this field have been made and have led to a lot of progress in understanding the molecular biology of HEV [36]. Several HEV isolates, such as the genotype 3 JE03-1760F strain, isolated from a Japanese patient with acute hepatitis E, and the genotype 4 HE-JF5/15F strain, isolated from a fulminant hepatitis patient with high viral load, have been used in various studies to decipher the molecular biology of HEV [6,37,38]. These two strains have been reported to replicate efficiently and generate reasonable viral titers when cultured in PLC/PRF/5 and A549, which are human hepatocellular carcinoma and human lung cancer cell lines, respectively [38,39]. More recently, the genotype 3 Kernow C1 (passage 6) strain, derived from a chronically HIV-HEV-infected patient and containing an in-frame insertion of 58 amino acids from the ribosomal protein S17, was shown to robustly replicate in Huh7 and HepG2/C3a liver cell lines, making it vital for cell culture studies. Furthermore, the insertion also widened host tropism [40,41].

Although cell culture systems have been instrumental in understanding some basic aspects of the molecular biology of HEV and the evaluation of novel antivirals, they are not suitable to help the scientific community in understanding the viral pathogenesis, including differences of HEV genotypes [5].

To dissect the in vivo characteristics of HEV infection and understand the viral pathogenesis, animal models are indispensable tools [42]. The circulation of HEV in a variety of animal species has led to the usage of several naturally occurring animal models. The ultimate animal model for HEV is fully immunocompetent, susceptible to distinct HEV strains that are able to cause infection in humans, and mimics the associated clinical disease. Ideally, this model is cheap, easy to handle and propagate, can be genetically manipulated, and comes with no or only limited ethical constraints. In this review we provide an overview of the currently available animal models for HEV. There is no model that fulfills all of the above-mentioned criteria, but each of the existing models have been requisite to a better understanding of this virus.

## 2. Non-Human Primate Model

### 2.1. Experimental Infection of Non-Human Primates

Non-human primates (NHP) are not a natural host for HEV, however they have been shown to be susceptible to experimental infection of genotypes 1, 2, 3, and 4 [43,44,45,46]. The first experiments using this model were performed on cynomolgus macaques. During the discovery of HEV as enterically transmitted non-A, non-B hepatitis in 1983, a human volunteer ingested stool samples derived from Afghan patients during a hepatitis outbreak. He developed symptoms of acute viral hepatitis whereupon his stool, containing virus-like particles (VLPs), was intravenously inoculated into cynomolgus macaques. Subsequently, it was confirmed that the agent was able to cause histopathological and enzymatical hepatitis in the macaques. Moreover, VLPs were excreted in the feces and a VLP-specific antibody response was observed [47].

After initial infection of cynomolgus macaques, also other NHP were used to study HEV. Purcell et al. performed a study to compare HEV infection in chimpanzees, cynomolgus, and rhesus macaques. Chimpanzees seemed to be slightly more susceptible to infection with HEV genotype 1 compared with cynomolgus and rhesus macaques, but it appeared that the same strain was more virulent in the macaques, shown by the elevation of liver enzymes. Moreover, rhesus monkeys were used to examine the difference in virulence between HEV genotypes. Genotype 3 HEV was significantly less virulent than human genotype 1 and 2 [44].

Pig-tailed macaques, vervets, owl monkeys, squirrel monkeys, and patas monkeys can all readily be infected with HEV, whereas studies in tamarins yielded mixed results as not all developed infection [48,49,50].

Infection of these animals is mostly performed intravenously. Oral inoculation has also been performed but failed in most trials. It was reported that oral inoculation of cynomolgus macaques required at least a 10,000-fold higher dose than intravenous inoculation in order to establish an active HEV infection [46]. The course of infection in NHP is similar to humans but with levels of viral shedding, liver enzyme elevation, and histopathologic changes in the liver that differ between the species.

### 2.2. NHP as Animal Model for HEV

Because of the abovementioned studies, it is accepted that chimpanzees, cynomolgus, and rhesus macaques are the most suitable models for HEV [51]. Chimpanzees and rhesus monkeys are susceptible to human HEV of genotypes 1 to 4. Cross-species infection studies showed that other zoonotic strains could also infect NHP. Chimpanzees and rhesus monkeys are susceptible to animal HEV of genotypes 3 and 4 [52]. Cynomolgus macaques could also be infected with a Chinese rabbit isolate of HEV and resulted in a typical HEV course with elevated liver enzymes, viremia, virus shedding in feces, and seroconversion [53]. Cynomolgus macaques were shown to be susceptible to an infectious gt 5 strain, generated by reverse genetics. This suggests that gt 5 may not only be confined to wild boar but has the ability to cause zoonosis [54]. Moreover, cynomolgus macaques could be infected with a gt 8 strain, isolated from Bactrian camels in China. Viremia, urinary, and fecal shedding of virus, elevation of liver enzymes, and seroconversion to anti-HEV IgM and IgG were all observed, as well as extra-hepatic replication. In one of the two tested animals, the virus persisted until week 25 after infection, which may present a chronic course in this animal. Further follow-up could not be achieved due to the unexpected death of the animal [55]. Attempts to infect cynomolgus and rhesus macaques with avian, rat, and ferret HEV have failed [56,57,58].

Next to testing the infectivity of different HEV genotypes, NHP models were also used to test infectious cDNA clones. Using RNA transcripts from variants of cDNA clones, it was confirmed that the 5′ cap is essential for infectivity of the virus [59]. Generation of such full-length functional clones provides opportunities to study the molecular biology of HEV in primates.

In an attempt to study HEV pathogenesis during pregnancy, 4 non-pregnant and 6 pregnant rhesus monkeys in the three different trimesters of pregnancy were inoculated intravenously with genotype 1 HEV. Unfortunately, the severe clinical course of fulminant hepatitis typically observed in pregnant women infected with HEV was not observed in the pregnant rhesus monkeys. In addition, the infection did not transmit from infected mothers to their offspring [60].

Cynomolgus macaques were also used to study chronic HEV and were persistently infected with a gt 3 HEV strain when being treated with tacrolimus, a potent calcineurin inhibitor immunosuppressant. Thereby, this situation mimics immunocompromised patients. Chronic infection in these animals was observed with a mild increase of liver enzymes serum levels, persistent RNA viremia, viral fecal shedding, and severe hepatic lesions [61].

Rhesus and cynomolgus macaques were also proven to be excellent models to evaluate the efficacy of potential HEV vaccines. Based on data observed in these animals, two HEV vaccine candidates containing recombinant HEV capsid antigen were applied to phase 2 and phase 3 trials in humans [43]. The first vaccine, HEV 239 (Hecolin®, China), contains a peptide encompassing 368–606 aa of ORF2 derived from a genotype 1 HEV Chinese strain expressed in *Escherichia coli* [62]. Rhesus macaques were vaccinated, after which they developed anti-HEV antibodies and were protected against challenge with homologous genotype 1 or heterologous genotype 4 HEV [63,64]. After extensive study of this vaccine in clinical trials with 100% protection for genotype 1 and 4 HEV, the vaccine was licensed in China in 2012, and is therefore the first commercial HEV vaccine [65,66]. Formal proof of its activity against genotype 2 and 3 HEV is, however, still lacking. Another recombinant HEV vaccine, consisting of a 56 kDa protein derived from capsid antigen ofthe gt 1 Pakistani Sar55 strain was expressed in insect cells from a baculovirus vector [62,67,68]. Several consecutive studies using this vaccine in cynomolgus and rhesus macaques showed the immunogenicity and efficacy in preventing HEV infection upon challenge with genotypes 1–3 HEV [67,68]. Subsequently, the vaccine was tested in human trials and showed 95% protection against genotype 1 HEV in military troops in Nepal [69].

### 2.3. Limitations of NHP Models

Although many studies have been carried out using NHP, they come with some disadvantages. As stated above, NHP are not a natural host of HEV and the observed clinical presentations of disease are sometimes limited. Moreover, chronic HEV as observed in immunocompromised patients infected with genotype 3 HEV has not yet been remodeled in primates. The use of these animals in future research is also limited due to ethical concerns, and invasive biomedical research in chimpanzees among other great apes is now severely restricted. Additionally, these animals may be difficult in operation with limited procedures, and come with a high cost [51].

## 3. Pig Model

### 3.1. Swine HEV and Course of Infection in Swine

Swine HEV was first discovered and characterized in 1997 in the United States and was, therefore, the first known animal strain of HEV [70]. It was shown that the majority of pigs older than 3 months were positive for anti-HEV IgG, indicating a high seroprevalence in commercial pig herds. Additionally, a prospective study was set up in these commercial herds to identify the HEV-related agent causing the seropositivity. According to previous sero-epidemiological data observed in pig herds, 18 of the 21 pregnant sows in this study tested positive for anti-HEV. Of those pregnant sows, 20 piglets were monitored for over 5 months to assess HEV infectivity. By 21 weeks of age, 16 of 20 studied piglets had seroconverted. Further genetical characterization revealed that swine HEV was closely related but distinct from human HEV of genotype 3 and 4, with a nucleotide sequence similarity of 79–80% and 90–92% on amino acid levels, respectively [70].

Since this initial discovery of swine HEV, it has become clear that swine represent a natural reservoir for HEV of genotype 3 and 4. Pigs become infected with the virus due to direct contact with an infected animal or through ingestion of feces-contaminated feed or water [71]. Experimentally, pigs are infected with swine HEV mostly by intravenous inoculation. Experimental infection via oral route has been difficult as detection of viremia, fecal shedding, or seroconversion to anti-HEV IgG could not always be observed [51,72]. After infection, pigs have transient viremia of about 1 to 2 weeks and shed the virus in large amounts in feces for 3 to 7 weeks. HEV infection in both naturally and experimentally infected pigs does not result in observable clinical disease or elevation in liver enzymes, however, several microscopic histopathological lesions can be observed, such as mild to moderate multifocal and periportal lymphoplasmacytic hepatitis and mild focal hepatocellular necrosis [71,73].

### 3.2. Pigs as an Animal Model for HEV

As natural reservoir for HEV of genotype 3 and 4, pigs serve as an important homologous animal model system. Specific-pathogen-free pigs can be readily infected with HEV of genotype 3 and 4 isolated from humans [74,75,76,77]. Moreover, HEV strains from other animals have been shown to cross the species barrier and cause infection in pigs. Inoculation with rabbit HEV, which is also a distant member of genotype 3, led to viremia and fecal virus shedding [78].

Shortly after the discovery of swine HEV and the identification of pigs as a homologous animal model, this model was used to study HEV replication. This resulted in the demonstration that the virus not only replicates in the liver, but also in extrahepatic sites, such as the small intestine, colon, and lymph nodes [79].

The pig model has also been used to assess the infectivity of capped full-length HEV RNA transcripts from infectious clones. These swine HEV infectious cDNA clones give an opportunity to study the basic biology of HEV and potential mechanisms of zoonotic transmission, as swine/human chimeric viruses can be constructed [80]. Thanks to this model it was also revealed that the initiation site of ORF3 is essential for viral infectivity in vivo and that the hypervariable region of ORF1 is unnecessary for infection and replication [81,82,83].

Vaccine studies have also been performed in pigs. Research showed that prior genotype 3 swine HEV infection prevented pigs from developing viremia and fecal virus shedding upon challenge with homologous swine HEV and heterologous human HEV strains. This finding is important as it may illustrate that a vaccine derived from one HEV genotype is sufficient to elicit protective immunity against other HEV genotypes [84]. Moreover, truncated recombinant capsid antigens derived from pig, rat, and chicken HEV strains could induce strong anti-HEV IgG responses in pigs and partially cross-protect against genotype 3 HEV [85].

More recently, a pig model mimicking chronic HEV was established by orally administrating a combination of 3 classical immunosuppressive drugs. Viral shedding in these pigs lasted 5–14 weeks longer than in immunocompetent pigs. Moreover, the pigs mimicked the immune response status of chronically infected human solid-organ transplant recipients. In the future, this model can be used to elucidate the mechanism of HEV pathogenesis and immune responses during chronic HEV infection, and to develop or evaluate antiviral treatments [86].

### 3.3. Limitations of the Pig Model

Although pigs have shown to be a useful model for HEV genotype 3 and genotype 4, they are not susceptible to genotype 1 and 2, which account for most of the clinical cases. Additionally, not all aspects of clinical disease are represented in pigs infected with genotype 3 and 4. Finally, pigs are also expensive, and due to their posture, not so easy to handle.

## 4. Rabbit Model

### 4.1. Rabbit HEV and Course of Infection in Rabbits

In 2009, the first rabbit HEV strain was isolated from a farm in China [87]. Since then, different rabbit strains across the globe have been found, suggesting that rabbits are a natural host for HEV [88,89,90,91,92]. After inoculation with different rabbit HEV strains, a typical course of acute HEV infection can be observed—virus is shed in the feces, liver enzymes are elevated, histopathological changes in the liver can be observed, HEV-specific antibodies appear, and the animals become viremic [93].

Interestingly, Han et al. showed that rabbits inoculated with the homologous CHN-BJ-rb14 HEV strain, had prolonged viremia and fecal shedding, indicating a chronic HEV infection [94]. Fecal shedding of the virus lasted for 9 months, and thereby exceeds the arbitrary 6-month definition of chronic infection [95]. In addition, liver histopathology showed chronic inflammatory cell infiltration and portal fibrosis [94]. In a subsequent study, a link between the viral dose and the development of chronic infection was suggested [96].

Rabbits experimentally infected with rabbit HEV also presented with extrahepatic replication in different tissues, such as the brain, heart, lungs, stomach, intestine, kidney, and placenta. Likewise, the chronically infected rabbits from the abovementioned study also presented with extrahepatic replication of HEV in the kidneys, suggesting that the observed lesions in this organ may have been induced by replication of the virus [96].

### 4.2. Rabbit as Animal Model for HEV

Rabbits are an attractive naturally occurring animal model for HEV research because of the viral genome sequence similarity between rabbit HEV and genotype 3 HEV that infects humans [97]. In fact, rabbit HEV was detected in 5 out of 919 HEV infected human subjects in France between 2015–2016 [98], suggesting zoonotic transmission. The strain isolated from these patients had an additional 93 nucleotides in the X-domain of ORF1, similar to viruses isolated from farmed and wild rabbits [91,98].

Rabbits can be successfully infected with human genotype 4 HEV, indicated by viral shedding in stool, elevation of liver enzymes, and histopathological changes in the liver [93,99].

Interestingly, when swine genotype 4 HEV, which also infects humans, was inoculated intraperitoneally into rabbits, it induced a productive infection that was evidenced by viral shedding in stool, viremia, and presence of HEV anti-sense RNA in the spleen, indicating extra-hepatic HEV replication [100]. Recently, Schlosser et al. succeeded in infecting rabbits with a wild-boar derived genotype 3 HEV. The animals shed virus in the feces and seroconverted four to five weeks post inoculation. Moreover, viral RNA was demonstrated in liver and gall bladder [101]. This data shows that rabbits could become handy in studying cross-species infection.

Another intriguing feature of HEV infection is the high morbidity and mortality among pregnant women [102]. Although the reason behind this is still unclear, immunological and hormonal changes during pregnancy may play a role [103]. In this line, Ahn et al. inoculated pregnant rabbits with rabbit HEV of strain KOR-Rb-1. The group observed that compared to the controls, the infected rabbits expressed significantly higher levels of aspartate transaminase, TNF-α, and IFN-γ, suggesting that these could possibly influence the outcome of HEV infection during pregnancy [104]. Elsewhere, Xia et al. reported that three out of the six experimentally infected pregnant rabbits died, while two of the remaining three miscarried [97]. Liver histopathology showed gross necrosis and infiltration of inflammatory cells. The presence of positive and negative sense RNA in the placental cells indicated vertical transmission [97]. Interestingly, Knegendorf et al. observed that HEV genotype 1 and 3 efficiently replicate and produce infectious viral particles in human-derived placental cells (JEG-3) [105]. Clearly, rabbit models open several doors to help us understand HEV pathogenesis during pregnancy.

HEV has been associated with some neurological extrahepatic manifestations. Tian et al. have used rabbits to investigate how the virus invades the nervous system. HEV RNA, as well as HEV ORF2 protein, could be detected in both brain and spinal cord. The CNS infections were also associated with pathological changes, including perivascular cuffs of lymphocytes and microglial nodules. These findings suggest that rabbits could also be a potential model to study HEV-associated neurological disorders [106].

Rabbits have also been used in HEV vaccine studies [99,107]. Cheng et al. administered 0, 10, and 20 µg of an HEV vaccine candidate p 179 to rabbits and then challenged them with a genotype 4 isolate (strain H4-NJ703). All the rabbits that received a 20 µg dose were protected from infection [99]. In another study, the HEV 239 vaccine was evaluated in rabbits for its efficacy at different doses. Results showed that administering two 10 µg doses of HEV 239 induced the best immune response in rabbits in comparison with two 20 µg doses or a single 30 µg dose [107]. In the work by Schlosser et al., rabbits immunized with recombinant rat HEV capsid proteins were protected from HEV genotype 3 infection [101]. From these three studies we can appreciate that rabbits present a good model for evaluating prophylactic vaccines and also possibly antiviral drugs. Although promising, more work needs to be done.

### 4.3. Limitations of the Rabbit Model

Rabbits are small animal models and have proven to be useful for cross-species infection, pathogenesis, pregnancy, and vaccine studies. However, it would be of great interest to establish such a kind of animal mimicking natural HEV-3 infection to help understand disease, and therefore provide novel insights into HEV replication. In regard to this, previous studies where rabbits were infected with human HEV of genotype 3 showed seroconversion but failed to show viral replication and shedding in feces [99,108]. In addition, rabbits are also resistant to human genotype 1 HEV [99].

## 5. Rat Model

### 5.1. Rat HEV and Course of Infection in Rats

Rat HEV was initially detected in *Rattus norvegicus* in Germany [109]. It is categorized under the species *Orthohepevirus C* and divided into three genetic groups [11,110]. When compared with human HEV, the nucleotide sequence similarity is approximately 60% [6]. Phylogenetic analysis indicates that rat HEV is much more related to ferret HEV [111]. *Rattus norvegicus* and *Rattus rattus* are among several rat species that are natural hosts of rat HEV. Over the years, rat HEV has been isolated from rats in Asia, the United States, and some countries in Europe [5]. In both wild and laboratory rats, rat HEV infection is not robust [57]. Purcell et al. showed that experimental infection of Sprague-Dawley rats was inconsistent and the magnitude of the infection was not sapid [57]. Although previously thought not to infect humans [57], it has recently been reported that rat HEV caused persistent hepatitis in a liver transplant patient in China [34]. This clearly shows the potential of zoonotic transmission.

### 5.2. Rat as Animal Model for HEV and the Associated Limitations

Establishing persistent HEV infection in laboratory rats had been challenging until recently when rat HEV, strain LA-B350, was reported to productively infect athymic nude rats [112]. In this work, Debing et al. observed that upon intravenous inoculation of LA-B350, viral RNA could be detected in stool as early as 4 days post-inoculation in all the infected animals. In fact, viral titers in serum reached about 4 × 10^6^ copies/mL [112]. Experimental infection of Wistar rats with mammalian HEV genotypes 1, 2, 3, and 4 has not been very successful. Maneerat et al. could demonstrate the presence of HEV RNA in stool of rats inoculated with a stool suspension of human HEV strain of undisclosed genotype [113]. In contrast, in the study of Schlosser et al., Wistar rats seemed resistant to intravenous inoculation of HEV-1 derived from a cynomolgus monkey, of HEV-3 derived from a domestic pig, and of HEV-4 derived from wild boar [101]. This shows that athymic nude rats might be the best surrogate model for studying HEV pathogenesis. However, lack of a functional immune system in these rats will prevent us from analyzing the impact the adaptive immune system plays in active pathological hepatitis [114].

## 6. Mouse Model

### 6.1. Mouse as Animal Model for HEV Infection

Most human pathogens do not naturally infect mice due to divergent host adaptation that has occurred over the years of evolution. To utilize mice as a model for studying human-specific pathogens, mice are genetically engineered to express specific human factors that render them susceptible to human pathogens [115,116]. Like other hepatitis viruses, human HEV does not naturally infect mice [101]. However, a few studies have recently shown that experimental infection of human liver chimeric mice with HEV genotype 1 and genotype 3 resulted in robust infection [117,118,119]. The hepatocytes of these mice are replaced by functional primary human hepatocytes, which can be achieved after introduction of severe liver disease [120]. First of all, this can be accomplished in mice carrying the mouse urokinase-type plasminogen activator (uPA) gene as a transgene under the control of the albumin enhancer/promotor (Alb-uPA). Overexpression of the uPA-gene in the liver results in severe liver injury allowing repopulation by transplanted human hepatocytes. To prevent rejection of the xenogeneic transplanted human hepatocytes, the uPA-mice first need to be backcrossed with an immune deficient mouse, such as the severe combined immunodeficient (SCID) mouse [121,122]. Using homozogous uPA-SCID mice, human hepatocytes integrate and repopulate up to 90% of mouse liver parenchyma [123]. A second option is to use mice carrying the fumarylacetoacetate hydrolase gene knockout (FAH^-/-^). Knockout of this essential enzyme taking part in the tyrosine catabolic pathway causes accumulation of hepatotoxic tyrosine metabolites, resulting in severe liver injury. These mice have been crossed with recombination activating gene 2 knockout (RAG2^-/-^) and IL-2 receptor γ-chain knockout (IL-2Rγ^-/-^) mice, then designated FRG mice [124]. Both of these two types of humanized mice have been used to study other hepatotropic pathogens, such as HCV, HBV, HDV, and *Plasmodium falciparum* [125,126,127,128].

Sayed et al. reported that when three uPA-SCID mice were inoculated with cell culture derived HEV genotype 3 Kernow C1-P6 (1.7 × 10^6^ IU/mouse), viral RNA could be detected in stool of two out of the three infected mice, with titers reaching around 6.2 × 10^4^ IU/mL [117]. Furthermore, when filtered stool suspension from an HEV genotype 1 (Sar-55) infected chimpanzee was intrasplenically inoculated into two mice, both tested positive for HEV RNA (stool and plasma) from week 1 post inoculation. In fact, the magnitude of the infection with genotype 1 was much more robust compared to genotype 3 [117]. Elsewhere, Van de Garde et al. showed that intravenous inoculation of fecal and liver derived inocula (HEV genotype 3 positive) into uPA^+/+^Nod-SCID-IL2Rγ^−/−^ mice resulted in productive infection [118].

We used humanized FRG mice to compare different routes of inoculation. Intrasplenic inoculation with a filtered patient stool suspension of HEV genotype 3 established infection in mice, with viral RNA in feces and delayed viremia. Inoculation with plasma from the same patient also established infection in one of two tested mice, however levels of fecal viral shedding were lower compared to inoculation with stool. Additionally, mice were also subjected to a genotype 1 strain (Sar-55) and RNA reached 10^4^ IU/mL from week 2 post-infection [129]. Oral inoculation in both humanized uPA-SCID and FRG mice did not result in a productive infection [117,129]. Humanized uPA-SCID mice have also been used to determine the infectivity of cell culture-derived virus preparations [8].

Next to uPA-SCID, uPA^+/+^Nod-SCID-IL2Rγ^−/−^, and FRG mice, other mice strains have been subjected to HEV, such as BALB/c mice and C57BL/6 mice. However, opposing results have been published using these wild type animals. Huang et al. reported successful infection of Balb/c nude mice with a swine feces-derived genotype 4 HEV. Infection was evidenced by HEV antigens and RNA in liver, spleen, kidney, jejunum, ileum, and colon, elevated liver enzymes, and anti-HEV IgG in sera [130]. Recently, the same group has also used regular Balb/c mice and infected them with a full-length swine HEV cDNA clone of genotype 4, constructed via reverse genetics. HEV RNA was detected in feces, serum, and tissues, such as liver, spleen, colon, and kidney [131].

Additionally, Sun et al. have succeeded in infecting Balb/c mice with a rabbit strain of HEV genotype 3 via fecal-oral route. The infected mice showed inflammatory lesions in liver, comparable with those observed in rabbits. HEV infection was evidenced with shedding of virus in feces, viremia, and seroconversion [132]. In contrast, a study using different C57BL/6 mouse strains (wild-type, IFNAR^-/-^, CD4^-/-^, and CD8^-/-^) and BALB/c nude mice failed to show infection of these animals when inoculated intravenously and orally with a wild-boar derived HEV genotype 3 strain [101]. Accordingly, Li et al. also failed to show infection of C57BL/6 mice when they were subjected to different HEV inocula (domestic-pig-derived HEV of genotype 3, wild-boar-derived HEV of genotype 4, and stool from a genotype 1 infected cynomolgus monkey). Viral RNA could not be detected in stool and serum samples from the inoculated animals [133].

### 6.2. The Limitations of Mice as HEV Animal Model

The above-mentioned data shows that human-liver chimeric uPA and FRG mice are very promising as small animal models for HEV research. However, the lack of a competent adaptive immune system in these mice makes them inadequate to study the immune-mediated pathogenicity of HEV [51]. Thus, the best option would be to utilize wild type immunocompetent mice, but these are still controversial given the discrepancies between the above highlighted studies. One alternative approach could be to humanize both the liver and immune system of the uPA-tg and FAH-KO models. Such dually engrafted animals have been implemented and already proved, to some extent, useful to study the human immune response upon hepatitis B infection [134,135].

Therefore, human liver chimeric mice seem to be the best option we currently have to study the dynamics of HEV infection and to test the efficacy of novel therapeutic approaches. The uPA-SCID model has already been validated for testing antivirals as a dose-dependent reduction of viral load was observed after ribavirin treatment, the first-choice treatment in humans [117,119]. The model has since been used to evaluate more experimental compounds, such as silvestrol [136].

## 7. Chicken Model

### 7.1. Avian HEV and Course of Infection in Chickens

Avian HEV was discovered in 2001 after isolation and characterization of the virus from the bile of chickens who presented with hepatitis-splenomegaly syndrome (HSS) [137]. Phylogenetic analyses revealed that the virus is genetically and antigenetically related and shares 60% sequence similarity with human HEV strains [138]. Since then, other diseases observed in chickens, such as big liver and spleen disease virus and hepatic rupture hemorrhage syndrome (HRHS), were shown to be caused by avian HEV [139,140]. Moreover, certain subclinical infections in chickens could also be attributed to avian HEV [141].

Chicken are the only known reservoir for avian HEV and the virus transmits easily between flocks via fecal-oral route [142]. Experimentally, chicken have been inoculated with avian HEV via intravenous or oronasal route. Upon infection, virus is shed in feces and viral RNA can be detected in serum, bile, and liver samples. Moreover, liver lesions characterized by subcapsular hemorrhages and enlarged lobe and periphlebitis. as well as lymphocytic phlebitis, can be observed in about 25% of infected chickens [143].

### 7.2. Chicken as an Animal Model and Its Limitations

The discovery of avian HEV and its relation to human HEV allows for the use of chicken as a homologous animal model to study HEV replication and pathogenesis. However, chickens are not susceptible to human or swine HEV and the observed clinical signs of infected do not correspond to the clinical signs in humans.

To gain insight into the morbidity and mortality associated with genotype 1 infections in pregnant women, chickens have been used to study vertical transmission. Guo et al. reported that infectious virus could be found in egg white from infected chickens. Unfortunately, no virus could be detected in the samples from hatched chicks, meaning that vertical transmission could not be completed [144].

Chickens have shown to be useful for vaccine studies, as immunization of chicken with an avian recombinant ORF2 capsid protein showed protective immunity against avian HEV infection, as none of the immunized chickens had detectable viremia, fecal virus shedding, or observable hepatitis lesions [145].

## 8. Other Small Animal Models

Alongside the described models, there are a few other animals that have proven to be useful for HEV research. One of those is the ferret. A ferret strain of HEV was first described in the Netherlands in 2010 and now appears to be widespread around the world [111,146,147]. Ferret HEV is distinct from human HEV, shares the highest similarity with rat HEV, and is assigned to the *Orthohepevirus C* species [111]. Therefore, the use of ferrets as an animal model to test antivirals or vaccines for humans is less relevant [26]. Experimental infection of ferrets leads to either subclinical infection, acute hepatitis, or persistent infection with detection of RNA in stool and sera, seroconversion, and significant elevation of alanine transaminase (ALT) [148]. The development of persistent infection was observed in 6 of 63 ferrets, and this is quite noteworthy as it developed naturally and not as a consequence of an immunosuppressive drug regimen [148]. Ferret HEV could not be replicated in Wistar rats, nude rats, or cynomolgus macaques [56].

Mongolian gerbils have also been shown to be susceptible to HEV. Gerbils could be experimentally infected with a genotype 4 swine HEV and a genotype 1 human strain. Infection with the former led to fecal viral shedding, elevation of liver transaminases, seroconversion, histopathological changes, and the virus could be detected in liver as well as sporadically in the kidney, spleen, and small intestine [149,150]. Infection with the human strain also led to fecal viral shedding and histopathological changes in liver, spleen, and kidney [151]. Gerbils have been used to study extrahepatic manifestations associated with HEV. It was reported that HEV RNA could be detected in the brain and spinal cord of genotype 4 infected animals, resulting in pathological changes in the brain and spinal cord. These observed pathological changes include degeneration and necrosis of neurons and Pirkinje cells, infiltration of inflammatory cells in ependymal epithelium and choroid plexus, and hemorrhages in choroid plexus, dorsal median septum, and central canal of the spinal cord. These are in accordance with the neurological manifestations observed in humans. As a consequence, gerbils prove useful for studying neurological disorders associated with HEV infection [106,152]. Furthermore, Soomro et al. examined the effect of HEV on testis tissue in genotype 4 infected animals. They showed that the structural and molecular changes upon infection were able to disrupt the blood-testis barrier and induce germ cell apoptosis [153]. The gerbil model was also used to evaluate the role of mast cells during infection, which appear to increase upon disease progression in various liver disorders. It was demonstrated that the amount of mast cells increased in the liver and small intestines upon infection, as well as higher expression of tryptase and serotonin, which indicate activation of the cells [154].

## 9. Conclusions

**Figure 1:**
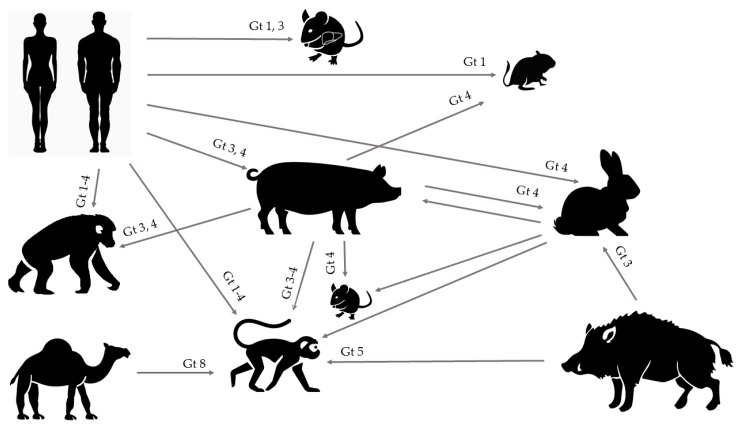
Successful experimental cross-species infections. A variety of human genotypes have been shown to cross the species barrier and infect human liver chimeric mice, gerbils, pigs, macaques, and chimpanzees. Genotype 3 and 4 isolated from pigs can cause infection in the non-human primate models, whereas BALB/c nude mice, gerbils, and rabbits were only susceptible to genotype 4 swine HEV. Rabbit HEV can replicated in pigs, BALB/c nude mice, and cynomolgus macaques, and rabbits themselves are prone to wild-boar derived genotype 3 HEV. Camel genotype 8 HEV and wild boar derived genotype 5 can infect cynomolgus macaques.

HEV is a worldwide underdiagnosed pathogen and the study of the underlying molecular mechanisms of HEV replication, pathogenesis, and more particularly, the host immune response, observation of chronic infection in immunocompromised patients, and severe outcomes in pregnant women have long been hampered due to an inefficient cell culture system and lack of convenient animal models. However, together with increasing recognition of this pathogen, improvements of both in vitro and in vivo models have emerged. The expanding host range of HEV has led to several new experimental and naturally occurring animal models, offering opportunities for future HEV research with strains from humans, pigs, wild boar, rabbits, and camels that are able to cause experimental cross-species infection (Figure 1). Although the ideal animal model mimicking all aspects of viral infection and disease progression in humans is yet to be identified, multiple of those aspects can be studied in some of the aforementioned models.
